# Immunotherapy with concurrent subcutaneous GM-CSF, low-dose IL-2 and IFN-*α* in patients with progressive metastatic renal cell carcinoma

**DOI:** 10.1038/sj.bjc.6600915

**Published:** 2003-04-29

**Authors:** N Verra, R Jansen, G Groenewegen, H Mallo, M J Kersten, A Bex, F A Vyth-Dreese, J Sein, W van de Kasteele, W J Nooijen, M de Waal, S Horenblas, G C de Gast

**Affiliations:** 1 Division of Immunology, Netherlands Cancer Institute, Plesmanlaan 121, 1066 CX Amsterdam, The Netherlands; 2 Departments of Medical Oncology of University Hospitals Maastricht and Utrecht, The Netherlands; 3 Division of Medical Oncology, Netherlands Cancer Institute, Plesmanlaan 121, 1066 CX Amsterdam, The Netherlands; 4 Division of Urology, Netherlands Cancer Institute, Plesmanlaan 121, 1066 CX Amsterdam, The Netherlands; 5 Division of Clinical Chemistry and Biostatistics, Netherlands Cancer Institute, Plesmanlaan 121, 1066 CX Amsterdam, The Netherlands

**Keywords:** renal cell carcinoma, immunotherapy, multicentre phase II study

## Abstract

The purpose of the study was to determine toxicity, efficacy and immunologic effects of concurrent subcutaneous injections of low-dose interleukin-2 (LD-IL-2), granulocyte–monocyte colony-stimulating factor (GM-CSF) and interferon-*α* 2b (IFN*α*) in progressive metastatic renal cell carcinoma. In a multicentre phase II study, 59 evaluable patients received two to six cycles of subcutaneous IL-2 (4 mIU m^−2^), GM-CSF (2.5 *μ*g kg^−1^) and IFN*α* (5 mIU flat^−1^) for 12 days per 3 weeks with evaluation after every two cycles. Cycles were repeated in responding or stable patients. Data were analysed after a median of 30 months follow-up (range 16–48 months). In 42 patients, the immunologic response was studied and related to response and survival. The main toxicity were flu-like symptoms, malaise and transient liver enzyme elevations, necessitating IL-2 reduction to 2 mIU m^−2^ in 29 patients, which should be considered the maximal tolerable dose. The response was 24% (eight out of 34, three complete response (CR), five partial response (PR)) in patients with metachronic metastases and 12% (three out of 25, 2CR, 1PR) in patients with synchronic metastases. Overall response was 19% (11 out of 59). Median survival was 9.5 months. All tested patients showed expansion and/or activation of lymphocytes, T cells and subsets, NK cells, eosinophils and monocytes. Pretreatment HLA-DR levels on monocytes and number of CD4^+^HLA-DR^+^ cells correlated with response. Pretreatment number of CD4^+^HLA-DR^+^ cells and postimmunotherapy levels of lymphocytes, CD3^+^, CD4^+^ and CD8^+^ T cells, but not of NK or B cells, correlated with prolonged survival. Immunotherapy with concurrent subcutaneous GM-CSF, LD-IL-2 and IFN*α* has limited toxicity, can be given as outpatient treatment and can induce durable CR. Response and survival with this form of immunotherapy seem to be more dependent on expansion/activation of T cells than of NK cells.

Metastatic renal cell carcinoma (mRCC) is insensitive to chemotherapy and only moderately sensitive to radiotherapy. As it is considered to be one of the most immunogenic tumours, immunotherapy has been studied extensively (Bukowski, 1999; Motzer *et al*, 2000). Currently, high-dose interleukin-2 (HD-IL-2)-based immunotherapy seems most promising as treatment for mRCC. Long-lasting complete responses (CRs) are induced in 5–10% of the patients (Fyfe *et al*, 1995; Bukowski, 1997; Negrier *et al*, 1998). Unfortunately HD-IL-2 therapy is rather toxic, requiring intensive care treatment in a considerable number of patients. A less toxic treatment, generally used in Europe as single-agent therapy, is subcutaneous (s.c.) interferon-*α* 2b (IFN-*α*) (Fossa, 2000). The combination of HD-IL-2 and IFN*α* did not induce more responses than either of the single-agent treatments alone (Bukowski, 1997; Negrier *et al*, 1998).

The mechanism of action of HD-IL-2 therapy in mRCC is not exactly known. The therapeutic effect can be exerted by either activated T cells or activated NK cells. In mRCC patients several immunologic defects have been described, comprising insufficient antigen-presenting cell numbers, suppressed function of dendritic cells (DCs) as well as T-cell function defects (Troy *et al*, 1998; Bukowski, 1999).

We combined s.c. low-dose IL-2 (LD-IL-2) with IFN*α* and granulocyte–monocyte colony-stimulating factor (GM-CSF) to activate all limbs of the immune system and to avoid the toxicity of intravenous (i.v.) HD-IL-2. These three cytokines together stimulate all cells known to be involved in the induction of antitumour responses: IL-2 stimulates T cells (Kolitz *et al*, 1987; Thompson *et al*, 1988), IFN*α* induces better effector cell functions and a higher expression of adhesion molecules and MHC class I on tumour cells (Knop, 1990; Luft *et al*, 1998), while GM-CSF gives proliferation and differentiation of DC (Ragnhammar, 1996). Therefore, the combination may correct most defects in the immune system.

After a phase I trial to determine the maximum tolerable dose (MTD) of the combination of these cytokines (de Gast *et al*, 2000), we started a multicentre phase II trial in the Netherlands with the MTD. We here report the results of that phase II study in 63 patients receiving daily s.c. injections of LD-IL-2, IFN*α* and GM-CSF for 12 days per 3 weeks. Apart from toxicity and efficacy, an important part of the study was to determine activation and expansion of immune cells in peripheral blood and to investigate the correlation of these parameters with response to therapy and survival.

## PATIENTS AND METHODS

### Patients

All patients entered into the trial, approved by the local ethical committees, had biopsy or cytologic proven progressive mRCC and had signed informed consent before therapy. Eligibility criteria further included age above 18 years, WHO performance score 0–2, ability to give informed consent, adequate bone marrow function (leucocytes >4.0 nl^−1^, platelets >100 nl^−1^) and adequate renal (creatinine <180 *μ*mol l^−l^) and liver function (transaminases, alkaline phosphatase <3× upper limit of normal (ULN), in case of liver metastases <5× ULN, normal bilirubin).

Exclusion criteria included serious cardiac, vascular, pulmonary or metabolic disease, pregnancy or lactation, systemic immunosuppressive treatment, including systemic steroids, previous or present autoimmune disease, aHIV antibodies and presence of brain metastases unless solitary and removed. Previous immunotherapy with IFN*α* was allowed but had to be stopped at least 2 months before the start. Previous radiotherapy for bone metastasis was allowed. Patients with only bone metastasis were excluded. Characteristics of the 63 patients are listed in Table 1

**Table 1:** Characteristics of the 63 eligible patients

*Age (years)*	
Median	58
Range	31–75
	
*WHO performance status*	
0	36
1	23
2	4
	
Male/female	44/19
Synchronic metastases	25
Metachronic metastases	38
	
*Sites of disease*	
Soft tissue only	3
Lung metastasis (±soft tissue)	36
Bone+others organs	19
Liver±other organs	9
Adrenal±lung	2
	
*No. of metastatic sites*	
1	23
2	25
>2	15

. In all, 38 patients had metachronic metastases (metastases that developed some time after nephrectomy) and 25 patients had their primary tumour still *in situ* at start of therapy (patients with synchronic metastases). A majority of patients had lung metastases and two or more sites of metastases.

### Treatment

Low-dose interleukin-2 (Aldesleukin, Proleukin^R^ 4 mIU m^−2^, Chiron BV Amsterdam, the Netherlands), GM-CSF (Molgramostim, Leukomax^R^ 2.5 *μ*g kg^−1^, provided by Schering-Plough, Maarssen, the Netherlands) and IFN*α* (Intron-A^R^, 5 mIU fixed dose, Schering-Plough, Maarssen, the Netherlands) were given as daily s.c. injections for 12 days in, respectively, abdominal wall, left leg and right leg every day at different places. Immunotherapy was initiated in the first cycle under controlled conditions in the hospital and continued at home after 2–3 days when the drug dosage was deemed safe for home administration. The remainder of the first cycle and following cycles (every 3 weeks) was completely given as outpatient treatment.

### Dose modification

Interleukin-2 dose was reduced to 2 mIU m^−2^ if grade 4 fever with hypotension, persistent severe malaise, diuretics insensitive weight gain >5% or grade 3 liver enzyme elevations did occur. Granulocyte–monocyte colony-stimulating factor had to be reduced by 50% if allergic symptoms persisted despite adequate treatment with antihistaminics. On the basis of leucocyte numbers in the blood after 7 days, GM-CSF was stopped immediately (if leucocytes >30 nl^−1^ on day 7), stopped after 9 days (if 25–30 nl^−1^), or continued for the full 12 days (if <25 nl^−1^ on day 7) in order to prevent excessive leucocytosis with eosinophilia. In case of any grade 3 CTC toxicity except fever and flu-like syndrome, medication had to be interrupted until resolution of that toxicity and the most probable causative agent reduced to 50% in the next cycle.

### Supportive measures

Prior to the start of immunotherapy, patients received 1/2 l of saline (NaCl 0.9%) intravenously and during immunotherapy Acetaminophen 1 g with the injections, 1 g at the start of chills and 500 mg tablets if necessary to a maximum of 4 g per 24 h. Metoclopramide was used to treat or prevent nausea. No systemic steroids were allowed unless absolutely necessary and NSAIDs were avoided in order not to suppress macrophage function.

### Evaluations

Prior to treatment, intravenous contrast enhanced computer tomography (CT) scans of chest, abdomen and pelvis were made, magnetic resonance imaging (MRI) of the brain and radioactive technetium scan of the bone was performed in addition to physical examination (PE) to determine the extent of the disease. In addition, an electrocardiogram and blood tests for haematology, liver and renal function and an HIV antibody test were performed.

Complete blood counts, differential WBC count, platelet count, liver and renal function tests were performed at start of treatment, after 1 week and at the end of immunotherapy (after 12 days) and after 3 weeks (before the next cycle). In addition, the absolute numbers of T cells (CD3, CD4, CD8), NK cells (CD3-CD16+56+), monocytes (CD14+) and B cells (CD19+) and of the activated cells (double staining with HLA-DR) were determined before treatment, at day 12 and day 23 with monoclonal antibodies on the FACS scan flow cytometer as described before (de Gast *et al*, 2000). Soluble IL2-receptor (sIL2R, Eurogenetics, Tessenderlo, Belgium) and sCD8 (T cell Diagnostics, Cambridge MA, USA) were assayed by ELISA as described before (de Gast *et al*, 2000).

### Response evaluation

Physical examination, CT scans and MRI of the brain were repeated after two cycles in week 7. Metastatic disease was quantified as the sum of products of perpendicular diameters of marker lesions. All measurable lesions on CT scans were used as marker lesions and up to 10 subcutaneous nodules were used as such. Responses were defined as CR: disappearance of all measurable disease; as partial response (PR): 50% or more reduction in measurable disease with no new lesions; as stable disease (SD): less than 25% increase or less than 50% decrease in measurable disease and no new lesions; or as progressive disease (PD): 25% or more increase in measurable disease or the appearance of new lesions, or nonreturn for evaluation because of deteriorating clinical condition. Responses must have been confirmed by another post-treatment evaluation 6 weeks later.

In case of PD, immunotherapy was stopped and palliative radiotherapy considered. In case of SD, another two cycles were given. In patients with regression, cycles were continued with a response evaluation after every two cycles. If CR was reached, two consolidation cycles were given. In patients with the primary tumour *in situ*, nephrectomy was performed and cycles with immunotherapy given within 6 weeks after recovery with response evaluation after every 2 cycles.

### Statistics

Survival curves were constructed using the Kaplan–Meier technique using the initiation of immunotherapy as starting point.

Levels of circulating cells in peripheral blood and of cytokines, at various time points and in various groups, were compared by Student's *t*-test. *P*-values <0.05 were considered significant.

## RESULTS

### Patients

In all, 63 patients started treatment. Four patients stopped treatment within 7 days because of rapidly PD (2), hypercalcaemia (1) or cardiac arrhythmia and decompensation (1). As shown in Table 2

**Table 2:** Response evaluation

	No. of patients			
		Synchronic metastases		
Response	Metachronic metastases	With Nx^a^	Without Nx	Total

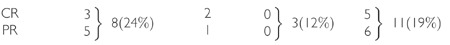				
SD	13 (1sCR)	9	0	22
PD	13	2	11	26

	34	14	11	59

a Nx=nephrectomy, CR=complete remission; PR=partial remission; SD=stable disease; PD=progressive disease, sCR=surgical CR.

, eight of the 34 evaluable patients with metachronic metastases achieved remission (24%, three CR, five PR) and 13 showed SD (38%). Four patients received IFN*α* for metastatic disease prior to treatment with concurrent immunotherapy. In two patients, a SD was reached, but no responses were seen. One CR patient relapsed in the bone after 8 months and died after 31 months. The two other CR patients are both in continued remission for 40+ months. One patient with an isolated lung metastasis after two cycles underwent a lobectomy and achieved a surgical CR, now maintained for 12+ months.

**Figure 1:**
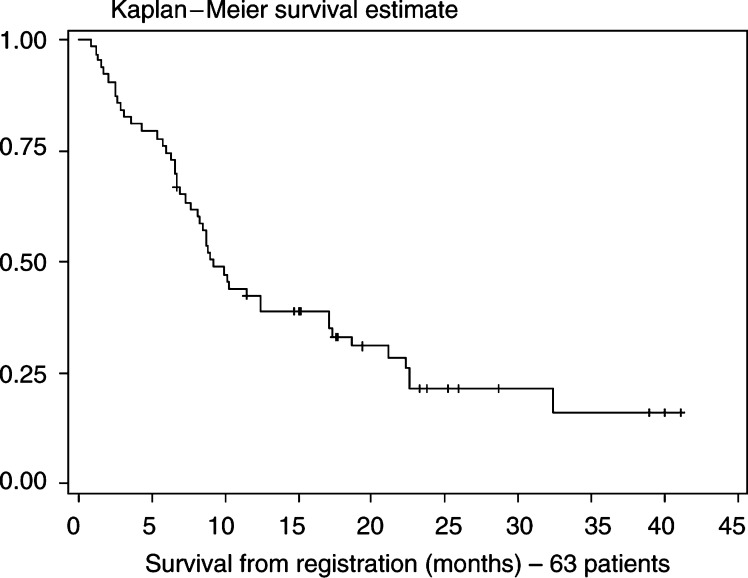
Median survival for all 63 registered patients was 9.5 months

Of the 25 patients with synchronic metastases, only 14 underwent nephrectomy and 11 patients underwent no nephrectomy, because of rapidly progressive disease ( Table 2). Two patients achieved CR after nephrectomy, of whom one got a relapse of his lung and pleural lesions after 4 months and one is still in continued CR for 15+ months. One patient with lung and adrenal metastasis achieved a PR. The response rate in the whole group was 11 out of 59 (19%) with five CR and six PR. Three of the five CR patients are in maintained remission (15+, 40+, 40+ months). Survival of all patients entering the study (*n*=63) is shown in Figure 1

.

### Toxicity

All patients developed flu-like symptoms with fever, chills, malaise and anorexia, which was only partly alleviated by Acetaminophen (Table 3

**Table 3:** Toxicity (NCI-CTC 2.0)

	Grade 2	Grade 3	Grade 4
1. Fever/chills	47	8	8
2. Fatigue	40	15	0
3. Liver function	9	5	0
4. Renal function	12	1	0
5. Allergic reactions	5	2	0
6. Cardiovascular	0	1	0
7. Haemoglobin	6	0	0
8. Platelets	0	0	0
9. Granulocytes	0	0	0

). Interleukin-2 was reduced to 2 mIU m^−2^ in eight patients with grade 4 fever with hypotension on the first day. As a result of persistent fatigue grade 3, IL-2 was reduced in another 15 patients after 1 week. In addition, IL-2 was reduced in the second cycle because of grade 3 liver function disturbances (only enzyme elevations) in five patients and because of grade 3 creatinine elevation in one. So in total, IL-2 was reduced from 4 to 2 mIU m^−2^ in 29 out of 59 patients, who received at least 1 full cycle of combined immunotherapy. A further reduction to 1 mIU m^−2^ of IL-2 was necessary in two patients because of persistent grade 3 fatigue. Allergic reactions grade 3 (angioedema) occurred in two patients, rapidly reacting to antiallergics and stopping of GM-CSF. One patient had cardiac arrhythmia and decompensation after 3 days, necessitating stopping of all treatment.

Leucocytosis with eosinophilia was seen in all patients during immunotherapy (median 22 nl^−1^, range 15–37 nl^−1^). Granulocyte–monocyte colony-stimulating factor was stopped because of excessive leucocytosis in only two patients.

None of the patients died of treatment-related toxicity or needed hospital admission.

### Immunologic evaluation

**Figure 2:**
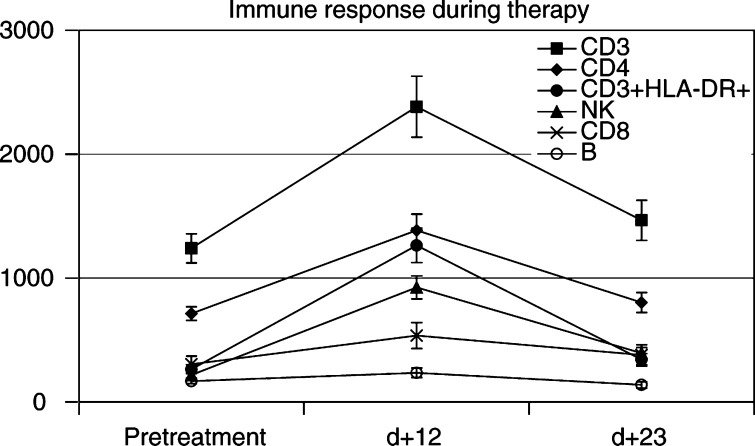
The number of T cells (CD3), activated T cells (CD3/DR), T helper cells (CD4), cytotoxic T cells (CD8) and of NK cells showed a significant increase after 12 days of immunotherapy, but not the number of B cells (CD19).

From all patients treated in the Antoni van Leeuwenhoek Hospital, Amsterdam (*n*=42) blood samples were taken during and after therapy to assess the effects of combined immunotherapy on immunologic parameters in peripheral blood. The parameters that were tested were lineage markers for T cells, B cells, NK cells and monocytes and number of eosinophils. To test the activation status, concomitant HLA-DR expression on lymphocytes and monocytes and sIL-2R and sCD8 levels in serum were examined. All markers, except the number of B cells, showed a significant increase on day +12 of therapy (Figure 2

and data not shown). At 11 days after cessation of the therapy most markers had returned to baseline values. In order to identify prognostic markers, patients with a response (CR/PR), SD or PD were studied for all the parameters. In Table 4

**Table 4:** Immunologic evaluation and response

Cell type pre^a^	Responders	SD	PD	Resp. *vs* PD
CD4^+^DR^+^	111±32^b^	113±40	57±8	*P*=0.05
CD4	870±169	658±47	696±98	NS
CD8^+^DR^+^	208±123	76±10	52±16	NS
CD8	625±301	236±22	215±34	NS
CD14	734±93	791±80	780±72	NS
HLA-DR level on monocytes	1059±167	722±84	556±77	*P*=0.01

a Cell type pre=cell type before immunotherapy;

b Cells nl^−1^ in peripheral blood, mean±s.e.; SD=stable disease, PD=progressive disease, …DR^+^=double fluorescence with HLA-DR, NS=nonsignificant *P*-value.

, the most relevant data are shown. The values of the patients with SD were generally found to be intermediate between the values of responders and PD patients. No significantly different markers were found for the CR/PR group *vs* the SD group as well as for the SD group *vs* the PD group. However, when the values of patients with CR/PR before immunotherapy were compared to those with PD, the responders had significantly higher numbers of activated CD4 T cells, but not of total numbers of CD4 T or (activated) CD8+ T in their peripheral blood before immunotherapy. Furthermore, the expression level of HLA-DR on monocytes was significantly higher in patients with CR/PR than in patients with PD. In contrast, the total number of monocytes did not differ between the two groups.

The patients monitored for immunologic markers were also stratified into two survival groups, based on the median survival: a prolonged survival group (survival 10 months or longer) and a short survival group (survival less than 10 months). As expected, the number of responders (seven out of 20) and SD patients (11 out of 20) was higher among the patients with prolonged survival than among the patients with short survival (one out of 22 responder and seven out of 22 SD). Only one prognostic marker was found when the pretreatment values were compared with survival. As shown in Table 5

**Table 5:** Immunologic evaluation and survival

		Long survival (*n*=20)	Short survival (*n*=22)	*P*-value
CD4^+^DR^+^	Pre^a^	130±34^b^	56±8	*P*=0.05
Lymphocytes	Post	2689±374	1777±122	*P*=0.02
CD3	Post	1897±350	1215±119	*P*=0.04
CD4	Post	1058±132	652±82	*P*=0.01
CD8	Post	606±190	249±31	*P*=0.03
NK	Post	459±69	359±53	NS
sIL-2R^c^	Post	15 049±2323	9447±1170	*P*=0.05
sCD8^c^	Post	889±155	641±92	NS

a Pre=pretreatment,

b cells nl^−1^ in peripheral blood; mean±s.e.;

c arbitrary ELISA units, NS=nonsignificant *P*-value.

, a significantly higher number of activated CD4 T cells was found in the prolonged survival group compared to the short survival group. Furthermore, comparing the values at the end of immunotherapy (day 12), five more markers were found. Total number of lymphocytes, of CD3, CD4 and CD8 T cells and levels of sIL-2R, but not of NK cells or levels of sCD8, correlated with survival.

## DISCUSSION

As RCC is known for its resistance against chemotherapy and for its immunogenic properties, cytokines have been used with various successes to treat this disease. The cytokines used in the current immunotherapy protocol, LD-IL-2, IFN*α* and GM-CSF, have been reported to induce antitumour activity to a greater or lesser extent (Sarna *et al*, 1987; Figlin *et al*, 1988; Vogelzang *et al*, 1993; Schiller *et al*, 1996; Bukowski, 2000; Fossa, 2000; Westermann *et al*, 2001). The combination of these three cytokines given simultaneously is based on the idea that expansion and activation of antigen-presenting cells (by GM-CSF), T cells (LD-IL-2 and GM-CSF) and effector cells (by LD-IL-2, GM-CSF, IFN-*α*), as well as an increase in immunosensitivity of the tumour cells (by IFN-*α*) is required for an optimal effect. Concurrent immunotherapy consisted of daily s.c. injections of 4 mIU m^−2^ IL-2, 2.5 *μ*g kg^−1^ GM-CSF and 5 mIU fixed dose IFN-*α* for 12 days per 3 weeks, a dose determined as the MTD in a phase I study (de Gast *et al*, 2000). However, in this phase II study IL-2 reduction to 2 mIU m^−2^ was necessary in 29 of the 59 patients because of grade 4 fever with hypotension (eight), severe fatigue (15), grade 3 liver enzyme elevations (five) or grade 3 renal function impairment (one). Therefore, we have to conclude that the MTD of this combination is IL-2 2 mIU m^−2^, GM-CSF 2.5 *μ*g kg^−1^ and IFN*α* 5 mIU fixed dose. With the lower dose of IL-2 outpatient treatment was possible without treatment-related mortality or hospital admission. In all, 59 evaluable patients showed an overall response rate of 19%, with 9% CR, 10% PR, 38% SD and 43% PD, which seems to be not different from HD-IL2 and maybe superior to IFN-*α* treatment concerning response.

In two recently published trials (Flanigan *et al*, 2001; Mickisch *et al*, 2001) nephrectomy followed by immunotherapy was found to be superior to immunotherapy alone with IFN-*α* with respect to survival in patients with synchronic metastases. In our series only 12 out of 25 patients with synchronic metastasis had a nephrectomy because of rapidly PD in the other 13 patients.

High-dose interleukin-2 therapy can elicit serious NK cell-mediated toxicity, necessitating intensive care treatment and causing up to 4% treatment-related mortality. In two larger studies (Fyfe *et al*, 1995; Negrier *et al*, 2000), an objective response rate of 15–19% was found, with 5 and 6% CRs, respectively. Of the complete responders, 75% remained disease free after 3 years. With our study, we aimed to develop a better tolerated treatment than HD-IL-2 i.v., which can be given as outpatient treatment and has a similar response rate. Response rate, percentage of durable CRs (three out of 59=5%) and survival seem not to be different from HD-IL-2 treatment, but tolerability seems better and our treatment could be given as outpatient treatment. However, a phase III study would be necessary to prove therapeutic equivalence.

Although we intended to activate and expand T cells and not NK cells with a low dose of IL-2 (and GM-CSF) to avoid toxicity, we found in all patients an increase in number and activation status of all lymphocytes tested, including NK cells, with the exception of B cells. As no vascular leak syndrome, because of excessive NK cell activation, was observed in any of the patient, the IL-2 dose was not too high. Two markers appeared to correlate with response. Both activated CD4 helper T cells (CD4^+^HLA-DR^+^) and HLA-DR levels on monocytes demonstrated a significant difference between responders and those with PD. Strikingly, neither CD8 cytotoxic T cell nor monocyte numbers showed a correlation with response. It is tempting to speculate that antigen-presenting cells and helper T cells that were already activated in some patients contributed to the response seen in these patients.

A more important aspect of the study was the measurement of activation of immune cells and its relation to survival. To evaluate the effect on survival, patients were divided into those with prolonged survival (⩾10 months) and those with short survival (< 10 months). Again, activated CD4 helper T cells were found to predict prolonged survival in the pretreatment values. Evaluating the values after 12 days of therapy, five other markers showed a significant difference between the two groups: total lymphocytes, total T cells (CD3^+^), CD4^+^ helper T cells, CD8^+^ cytotoxic T cells and sIL-2R. No correlation with NK cells was seen in either response or survival groups, indicating that the effects of combined immunotherapy are not NK cell, but T cell and monocyte mediated.

Our trial is the first in which markers for response and survival of IL-2-based therapies are reported for mRCC. In a study reported by Westermann *et al* (2001), IL-2 and IFN-*α* were added sequentially to GM-CSF in doses comparable to our scheme. Although they also found a general increase in lymphocytes, the number of treated patients was too small to distinguish between responder groups. In a trial with LD-IL-2 i.v., 25 patients were treated and no correlation with response could be found for any of the lymphocyte populations tested (Favrot *et al*, 1990). In several other studies in which the combination of IL-2 and IFN-*α* was used, the trials were too small to determine a correlation with response to treatment (Bukowski, 1997). However, in a large trial with HD-IL-2 therapy for metastatic melanoma, responding patients were found to have a significant higher maximum lymphocyte count immediately after therapy (Phan *et al*, 2001). Notably, in that trial only total lymphocyte numbers were determined and no differentiation was made between T-cell and NK-cell numbers. This indicates that patients responding to immunotherapy may be identified by analysis of peripheral blood samples. The correlation between immunological markers and survival should first be confirmed in a prospective study. As our series was limited, a multivariate analysis was not possible to study whether the immune parameters added anything over the use of known clinical parameters determining the presence of low, intermediate or high risk for survival (Zisman *et al*, 2002).

Immunotherapy protocols are thought to be most effective if smaller amounts of tumour are present, as indicated by the patient groups that respond best to cytokine immunotherapy, for example, good performance status, prior nephrectomy, lung metastases only, few metastatic sites (Mani *et al*, 1995). This might indicate that this protocol could be more effective in a perioperative setting in which nearly all tumour can be removed. In a perioperative immunotherapy protocol, it is also possible to look at the effects of immunotherapy at the site of the tumour and to relate those results with the effects in peripheral blood. Especially the activation and attraction to the tumour site of DCs and T cells, in relation to the effects on peripheral blood, may give important insight into the kinetics of cells of the immune system. Such a trial, which is currently ongoing in our hospital, may give us more understanding in how the antitumor effect is achieved and may enable us to further improve upon cytokine immunotherapy.
